# Gender-Specific Cardiovascular Reactions to +Gz Interval Training on a Short Arm Human Centrifuge

**DOI:** 10.3389/fphys.2018.01028

**Published:** 2018-07-31

**Authors:** Zeynep Masatli, Michael Nordine, Martina A. Maggioni, Stefan Mendt, Ben Hilmer, Katharina Brauns, Anika Werner, Anton Schwarz, Helmut Habazettl, Hanns-Christian Gunga, Oliver S. Opatz

**Affiliations:** ^1^Center for Space Medicine and Extreme Environments Berlin, Institute of Physiology, Charité-Universitätsmedizin Berlin, Berlin, Germany; ^2^Department of Biomedical Sciences for Health, Università degli Studi di Milano, Milan, Italy; ^3^Central Medical School, Monash University, Melbourne, VIC, Australia

**Keywords:** artificial gravity, gender, short arm human centrifuge, cardiovascular deconditioning, artificial gravity training, countermeasure

## Abstract

Cardiovascular deconditioning occurs in astronauts during microgravity exposure, and may lead to post-flight orthostatic intolerance, which is more prevalent in women than men. Intermittent artificial gravity is a potential countermeasure, which can effectively train the cardiovascular mechanisms responsible for maintaining orthostatic integrity. Since cardiovascular responses may differ between women and men during gravitational challenges, information regarding gender specific responses during intermittent artificial gravity exposure plays a crucial role in countermeasure strategies. This study implemented a +Gz interval training protocol using a ground based short arm human centrifuge, in order to assess its effectiveness in stimulating the components of orthostatic integrity, such as diastolic blood pressure, heart rate and vascular resistance amongst both genders. Twenty-eight participants (12 men/16 women) underwent a two-round graded +1/2/1 Gz profile, with each +Gz phase lasting 4 min. Cardiovascular parameters from each phase (averaged last 60 sec) were analyzed for significant changes with respect to baseline values. Twelve men and eleven women completed the session without interruption, while five women experienced an orthostatic event. These women had a significantly greater height and baseline mean arterial pressure than their counterparts. Throughout the +Gz interval session, women who completed the session exhibited significant increases in heart rate and systemic vascular resistance index throughout all +Gz phases, while exhibiting increases in diastolic blood pressure during several +Gz phases. Men expressed significant increases from baseline in diastolic blood pressure throughout the session with heart rate increases during the +2Gz phases, while no significant changes in vascular resistance were recorded. Furthermore, women exhibited non-significantly higher heart rates over men during all phases of +Gz. Based on these findings, this protocol proved to consistently stimulate the cardiovascular systems involved in orthostatic integrity to a larger extent amongst women than men. Thus the +Gz gradients used for this interval protocol may be beneficial for women as a countermeasure against microgravity induced cardiovascular deconditioning, whereas men may require higher +Gz gradients. Lastly, this study indicates that gender specific cardiovascular reactions are apparent during graded +Gz exposure while no significant differences regarding cardiovascular responses were found between women and men during intermittent artificial gravity training.

## Introduction

Human space exploration inherently leads to micro-gravity (micro-g) exposure, which induces changes in cardiovascular functioning. The sum of physiological adaptations that occur during exposure to micro-g is known as cardiovascular deconditioning (Komorowski et al., 2016). Changes that occur comprise of cephalic fluid shifts, which lead to increases in cardiac output and myocardial atrophy (Norsk et al., 2015) with resulting decreases in blood volume (Agnew et al., 2004). Further changes that have been recorded are reductions in cardiac diastolic function (Convertino and Cooke, 2005), heart rate (Verheyden et al., 2009), and drastic reductions in systemic vascular resistance (Norsk et al., 2015). All of these adaptations contribute to a 50% reduction in baroreflex functioning (Antonutto and di Prampero, 2003; Beckers et al., 2009), which can lead to orthostatic intolerance (OI) upon return to Earth or any other gravitational environment (Lee et al., 2015). This is characterized by the inability of the neurohumoral reflex, namely increases in heart rate and vasoconstriction, to maintain adequate mean arterial pressure. Additional evidence also suggests that women astronauts undergo profound endothelial dysregulation during gravitational unloading (Demiot et al., 2007), which may contribute to the higher incidence of OI in women astronauts upon returning to Earth (Waters et al., 2002; Wenner et al., 2013).

Research in effective cardiovascular deconditioning countermeasures for both genders is imperative and is as a goal of the Human Research Roadmap put forth by NASA, as well as the EU (Aubert et al., 2016; Vernikos et al., 2016). A countermeasure system that can potentially offset micro-g induced cardiovascular deconditioning is the implementation of artificial gravity (AG) exposure via short arm human centrifuge (SAHC). AG via SAHC creates a gravitational vector along the z-axis of the body thereby stimulating the baroreflex system to induce upsurges in cardiac and vascular resistance activity (Moore et al., 2005). Several Earth-based studies have provided evidence that AG training can improve tolerance against OI, and that benefits of short intermittent AG exposure surpass those of continuous static AG exposure (Stenger et al., 2007, 2012; Young and Paloski, 2007; Goswami et al., 2015a; Clément et al., 2016; Zhang et al., 2017). Additionally, there is also evidence to suggest that short intermittent AG exposure improves tissue oxygenation (Marijke et al., 2017). Much like the benefits high-intensity interval training provides for maintaining aerobic fitness (Gibala et al., 2012; Milanović et al., 2015), AG training could prove to be an effective counter-measure against OI via short duration and intense training intervals. However, more research must be conducted using Earth-based AG protocols prior to testing in microgravity environments.

The limited number of AG studies performed to date, have primarily involved research in men, and have focused on orthostatic stability upon re-introduction of 1G conditions, and not so much on cardiovascular responses during the AG training itself. The cardiovascular responses in women during SAHC AG exposure have not been thoroughly documented and no studies have specifically have recorded and analyzed gender specific cardiovascular reactions active during an intermittent AG exposure. It has yet to be determined whether women and men exhibit similar or diverging cardiovascular responses during an identical AG protocol. A thorough comparison of gender specific cardiovascular responses during AG is important in order to verify its effectiveness on eliciting the cardiovascular responses needed to overcome an OI event. There is also substantial evidence indicating that cardiovascular functioning differs between men and women (Evans et al., 2001; Hart et al., 2009; Hart and Charkoudian, 2014), particularly cardiac and vascular resistance activity, with women tending to exhibit greater cardiac activity, whereas men tend to respond with heightened vascular resistance activity during orthostatic stress (Shoemaker et al., 2001). In addition, women have exhibited a greater gravity-dependent baroreflex sensitivity than men during orthostatic stress, which leads to profound differences in cardiovascular responses during exposure to an equal level of orthostatic stimulus (Drudi and Grenon, 2014). Prior to deployment of any AG protocol for manned space crews, it must be determined what +Gz gradient is required in order to elicit significant increases in cardiac and vascular resistance in both women and men, while minimizing any OI event during the exposure. Therefore, this study implemented a graded +Gz interval training (GIT) via SAHC in order to establish whether the cardiovascular systems involved in maintaining orthostatic integrity can effectively be stimulated amongst men and women, and to examine if any gender specific cardiovascular responses became apparent.

## Methods

### Subjects

Twenty-eight healthy, Caucasian civilian subjects gave their written informed consent to participate freely in this experiment, which took place at the German Aerospace Institute (DLR), at the European Space Agency (ESA) short arm human centrifuge (SAHC) test facility in Cologne, Germany. The first 13 subjects were tested in the fall of 2012, while the remaining 15 were studied in summer of 2015. The subject pool consisted of 16 women and 12 men who were matched for age (28.4 ± 5.3 years). Screening comprised of a medical questionnaire and a physical examination performed by an independent general physician who was not involved in the study. This screening examination included a resting ECG, a Schellong test to screen for orthostatic susceptibility, and cycle ergometry to determine baseline cardiovascular fitness. Upon completion of the screening, no subjects were excluded, nor had any history of cardiovascular, metabolic, or neurological diseases. Furthermore, it was ensured that none were commercial or military pilots, professional or elite athletes. This study was carried out in accordance with the recommendations of the Medical Ethics Committee of Nordrhein-Westfalen, Germany (Aerztekammer Nordrhein, Düsseldorf, Germany) in accordance with the Declaration of Helsinki. The protocol was approved by the Medical Ethics Committee of Nordrhein-Westfalen, Germany (Aerztekammer Nordrhein, Düsseldorf, Germany).

### Study protocol

The SAHC (SAHC-TN-007-VE, QinetiQ Company, Antwerp, Belgium) used for this study has a +Gz range of +0.1 to +5.0, an acceleration rate of maximal +0.4Gz/s, a maximum radius of 2.82 m and a maximum RPM of 40. The ESA-SAHC has been extensively used for +Gz training and various studies in the field of gravitational physiology (Zander et al., 2013; Frett et al., 2015).

**Figure 1:**
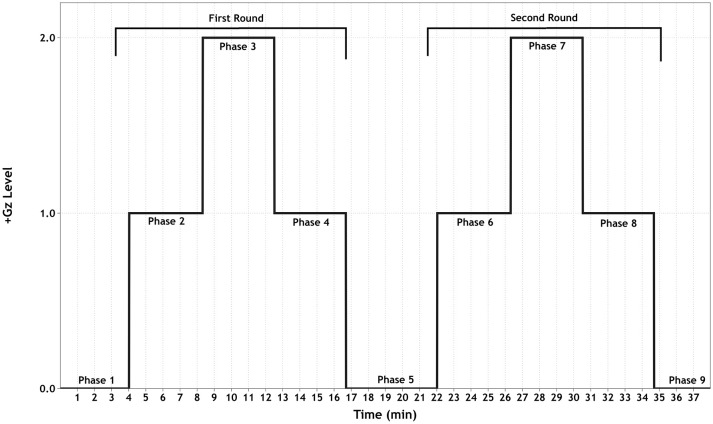
Schematic of the +Gz interval training (GIT) protocol. Each phase (1–9) is labeled accordingly, indicating the corresponding +Gz level over time in minutes.

The +Gz profile (see Figure 1) consisted of an initial baseline for 15 min followed by two identical +1/2/1Gz graded acceleration/deceleration rounds, composed of 3 phases each. These 2 rounds were separated by a +0Gz phase. Except for phase 1 (P1, 15 min) and P5 (5 min), each phase lasted 4 min. Acceleration/deceleration between each phase lasted 15 s. For the first 5 min of P1, the SAHC was not rotated. This was done to ensure good signal quality. For the next 10 min, the SAHC was slowly rotated at 5 RPM. During +1Gz phases (P2, P4, P6 and P8), the gravitational vector along the body was as follows: head +0.3Gz, mediastinum +0.5Gz, and feet +1Gz with an approximate RPM of 16. During +2Gz phases (P3 and P7), the gravitational vector along the body was +0.6Gz for the head, +0.9Gz for the mediastinum and +2.0Gz for the feet with an approximate RPM of 26.4. During P5, the SAHC was rotated at 5 RPM.

The purpose of implementing a graded +Gz protocol was to reduce the occurrence of OI due to rapid inductions of +2Gz. Therefore, the first +1Gz phase (mild hyper-gravity) was used to reduce any sudden occurrence of an OI event and to prime the baroreflex system for a moderate gravitational stress stimulus. The +2Gz phases induced moderate +Gz and the desired cardiovascular reactions. A reduction back to +1Gz allowed for mild +Gz exposure, with the addition of absorbing any carry-over effects of the +2Gz phase. P5 allowed for a return to baseline conditions before starting the second round. The two rounds of +Gz were set up in order to ascertain whether these reactions could be replicated, adding to the principle of interval training.

In case of signs of orthostatic intolerance (OI) during the GIT, the +Gz level was reduced or the protocol was terminated completely. These included perfuse sweating, confusion, dizziness, nausea, a narrowing of the visual field or the presence of hemodynamic decompensation criteria, such as new-onset ECG abnormalities, an abrupt drop in MAP of >20 mmHg or a critical narrowing of the pulse pressure. Additionally, each subject could press a “panic button” if they wished to abort the test. Direct verbal and visual contact was maintained between the flight physician and the test subject via camera and microphone.

#### Cardiovascular recording

Mean arterial blood pressure (MAP, mmHg) and pulse pressure waves were monitored continuously and non-invasively via finger plethysmography (Portapres®, Finapres Medical Systems BV monitoring, Amsterdam, the Netherlands). The collected blood pressure waves were analyzed with Beat-scope® software. Systolic and diastolic blood pressure (SBP, mmHg and DBP, mmHg respectively) as well as stroke volume (SV, ml) were extracted from the continuous blood pressure waveform. Heart rate (HR, bpm) was recorded via a 3-lead ECG. Cardiac output (CO, L/min) and systemic vascular resistance (SVR, mmHg·min/l) were calculated via the Wesseling formula (Veerman et al., 1995). To control for significant baseline anthropometric gender differences, CO, SV, and SVR were converted to cardiac index (CI L/min/m^2^), stroke volume index (SVI ml/m^2^) and systemic vascular resistance index (SVRI, mmHg·min/l/m^2^).

### Data and statistical analysis

All recorded data was synced according to phase and grouped according to gender. The last 60 s from each phase were averaged and used for the statistical analysis. This time section was chosen primarily to provide continuity with a previous study based on the first part of the same experiment, (Habazettl et al., 2016). In that study, microvascular and cardiovascular responses from the last 60 s of each phase were analyzed in order to highlight the maximal response. Furthermore, cardiovascular parameters in most subjects showed oscillation patterns during the first 2–3 min of each phase, thereby exhibiting high variability thus not allowing for an accurate assessment of cardiovascular reactions. Secondly, the last 60 s were used with the goal of reflecting maximal cardiovascular response as well as steady state without the interference of acute changes in +Gz transitions. Diaz Artiles et al. (2016) recorded the above-mentioned cardiovascular trends during an SAHC study, where it was shown that the participants reached maximal response or steady state during the last 60 s of SAHC exposure and the variability during this period was at a minimum.

Statistical analysis of cardiovascular reactions during the GIT were done via repeated measures ANOVA using phase comparisons from baseline as a within and gender as a between subject factor. To test for equality of variance between men and women the Levene's test was employed. Assumption checks were made using a Mauchly's test of Sphericity followed by Greenhouse-Geiser corrections. Effect size per cardiovascular parameter was calculated via partial Eta squared test. This was done for the whole group as well as independently for women and men. Changes from baseline were further analyzed using *t*-tests with Holm-Sidak *post-hoc* correction. To assess for differences in baseline, a student's *t*-test was used. All data is presented as mean and ± standard error of the mean (SEM). The level of statistical significance was defined at alpha = 0.05. Cardiovascular parameter synchronizing and averaging was performed using “R” statistical environment version 3.2.5 (R Core Team, 2013). Inferential statistics were performed using SPSS Statistics 23 (IBM, New York, USA) and JASP Version 0.8.2 for Mac OS (JASP Team, 2018). Graphics were created using Data Graph 4.2 software for Mac OS (Visual Data Tools, Inc., 2013).

## Results

From the 28 subjects, 23 (11 women and 12 men) completed the GIT without any interruption. All five non-finishers (NF) were women and will be referred to as womenNF. One woman exhibited an OI event in both +2Gz phases, however she endured all +1Gz phases. Three other women experienced an OI event during P3, two of which requested to terminate their GIT exposure at that point. One went on to complete P4 before terminating her exposure. Another woman experienced an OI event during P3, however she completed all subsequent phases, and P7 at a reduced +Gz level (1.5+Gz instead of +2Gz).

### Baseline

Anthropometric data are represented in Table 1. Height, weight and BSA were all significantly greater amongst men than women who finished the GIT (*p* < 0.05). The womenNF were significantly taller than finisher women (*p* < 0.05). Baseline cardiovascular values were obtained from the last 60 s of P1 and are represented in Table 2. The womenNF exhibited significantly greater MAP, DBP, and SVRI (*p* < 0.05), and non-significantly greater SBP (*p* = 0.054) than women who finished the GIT. There were no significant differences found between men and women who finished the GIT regarding baseline cardiovascular parameters. However, men had slightly higher DBP and SVRI at baseline, whereas women exhibited a higher HR.

**Table 1:** Mean and SEM baseline anthropometric data for women and men.

	**Men (*n* = 12)**	**Women (*n* = 11)**	**WomenNF (*n* = 5)**
Age (years)	29.0 ± 1.46	28.3 ± 1.90	27.4 ± 0.84
Height (cm)	178.1 ± 1.76^*^	168.8 ± 1.63	176.1 ± 0.59^#^
Weight (kg)	76.0 ± 1.10^*^	65.5 ± 2.36	67.3 ± 0.79
BMI (kg/m^2^)	24.0 ± 0.35	23.0 ± 0.66	21.6 ± 0.18
BSA (m^2^)	1.90 ± 0.02^*^	1.70 ± 0.04	1.83 ± 0.01

* *Denotes a significantly greater value in men compared to women (p < 0.05)*.

# *Denotes a significantly greater value in womenNF compared to the finisher women (p < 0.05)*.

**Table 2:** Mean and SEM baseline cardiovascular data for women and men.

	**Men (*n* = 12)**	**Women (*n* = 11)**	**WomenNF (*n* = 5)**
MAP (mmHg)	80.8 ± 4.05	77.7 ± 3.79	101.6 ± 8.05^*^
DBP (mmHg)	64.2 ± 3.80	58.1 ± 2.99	79.6 ± 6.20^*^
SBP (mmHg)	126.4 ± 4.17	126.1 ± 7.17	154.6 ± 12.55
SVRI (mmHg*min/l/m^2^)	25.8 ± 2.26	21.9 ± 1.21	30.3 ± 1.41^*^
HR (bpm)	63.7 ± 3.75	68.5 ± 3.75	70.8 ± 6.67
CI (l/min/m^2^)	3.30 ± 0.17	3.60 ± 0.19	3.30 ± 0.23
SVI (ml/m^2^)	51.1 ± 2.27	52.0 ± 1.48	47.4 ± 3.70

* *Denotes a significantly greater value in womenNF compared to the finisher women (p < 0.05). The p-value for SBP between womenNF and finisher women was nearly significant (p = 0.053)*.

### Cardiovascular reactions

**Figure 2:**
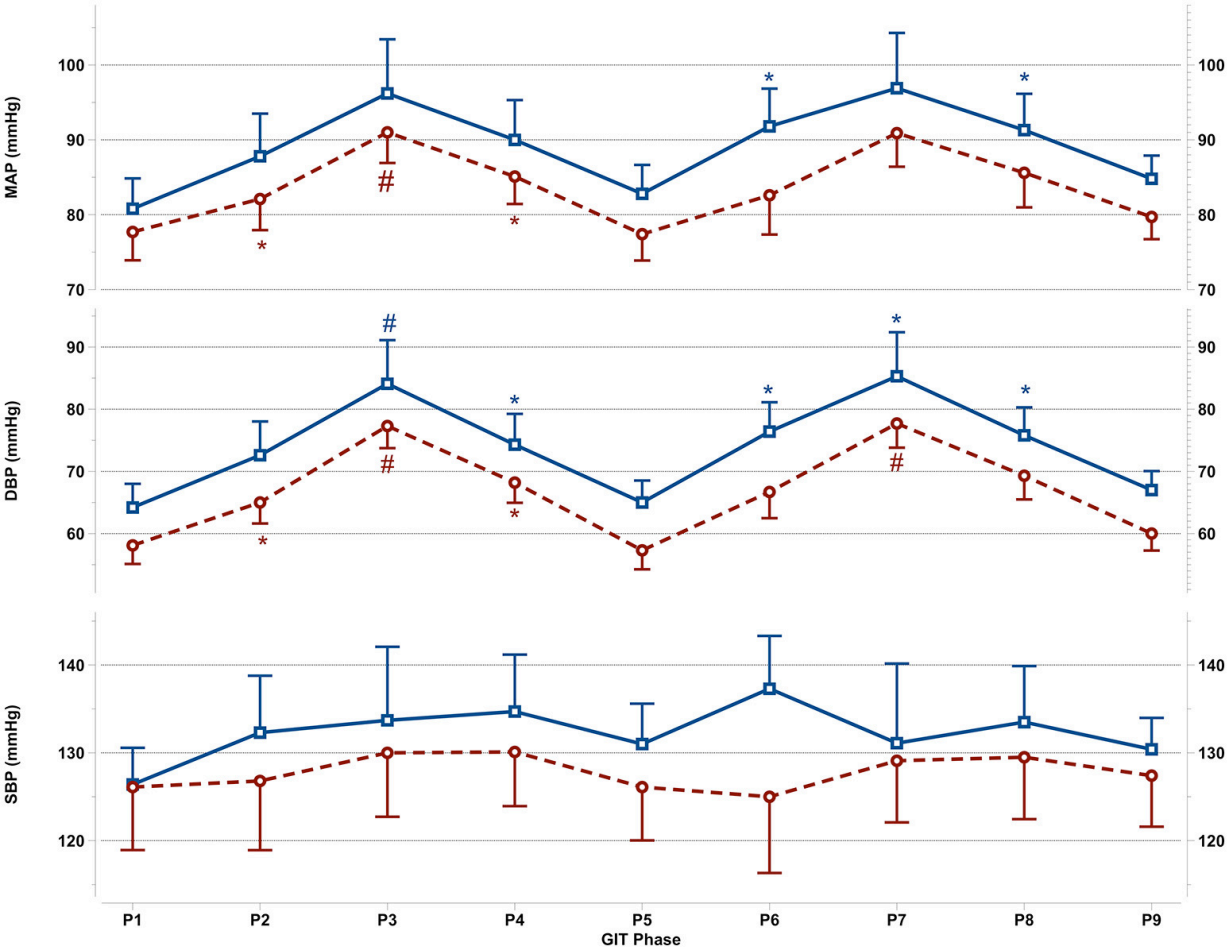
Mean and SEM for central blood pressure reactions of the last 60 s per +Gz phase from top to bottom: MAP, mean arterial pressure DBP, diastolic blood pressure, and SBP systolic blood pressure during GIT for men (blue squares, solid lines *n* = 12) and women (red circles, spaced lines *n* = 11). *Denotes a significant change from baseline P1 (*p* < 0.05), and ^#^indicates a significant change between +2Gz and +1Gz phases (*p* < 0.05). GIT phase nomenclature is indicated along the x-axis (P1-P9).

**Figure 3:**
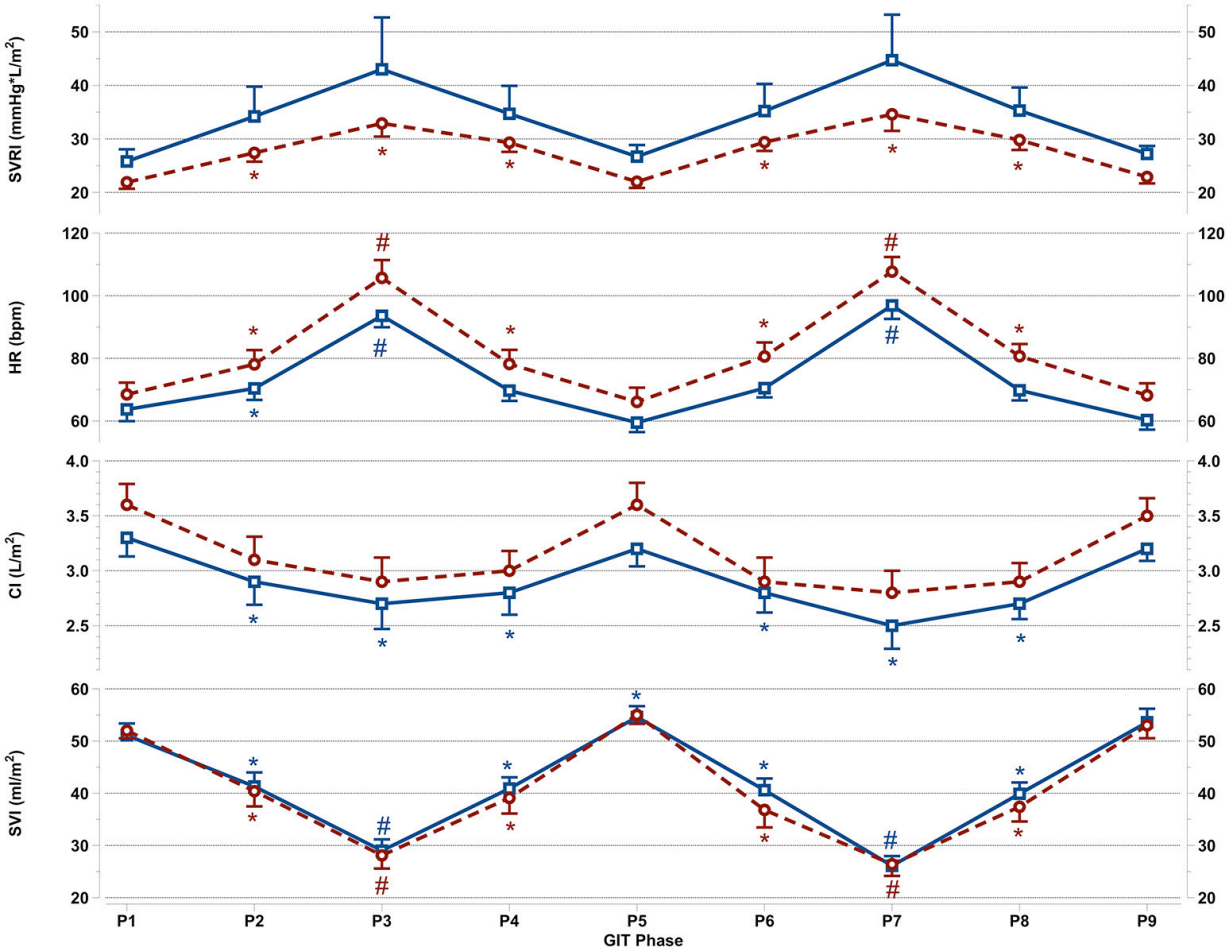
Mean and SEM for vascular resistance and cardiac reactions of the last 60 s per +Gz phase from top to bottom: SVRI systemic vascular resistance, HR heart rate, CI cardiac index, and SVI stroke volume index for men (blue squares solid lines *n* = 12) and women (red circles spaced lines *n* = 11) during GIT. *Denotes a significant change from baseline P1 (*p* < 0.05), and ^#^indicates a significant change between +2Gz and +1Gz phases (*p* < 0.05). GIT phase nomenclature is indicated along the x-axis (P1-P9).

All cardiovascular parameters passed the test of normality (p > 0.05). The respective effect sizes, F-statistics and *p*-values for each parameter are summarized in Table 3. The cardiovascular responses for women and men during GIT are displayed as line plots in Figures 2, 3. Across the seven observed cardiovascular parameters, there was a significant main effect of +Gz (*p* < 0.05) across all phases for women and men, with the exception of SBP (Table 3). No significant differences were found amongst cardiovascular parameters between women and men.

**Table 3:** Partial Eta-squared effect sizes, F-statistics, and *p*-values with Greenhouse-Geiser sphericity corrections per cardiovascular parameter for women, men, and all subjects for main effect of phase.

		**Effect size partial Eta-squared per parameter**	**F-Statistics per parameter**	***p*-value per parameter**
MAP (mmHg)	Women	0.359	5.61	0.012
	Men	0.387	6.93	0.012
	All	0.368	12.22	< 0.001
	Women vs. Men	0.04	0.804	0.380
DBP (mmHg)	Women	0.646	18.3	< 0.001
	Men	0.553	13.6	0.001
	All	0.591	30.36	< 0.001
	Women vs. Men	0.071	1.61	0.218
SBP (mmHg)	Women	0.037	0.39	0.748
	Men	0.092	1.11	0.343
	All	0.039	0.85	0.477
	Women vs. Men	0.013	0.273	0.607
SVRI (mmHg*min/l/m^2^)	Women	0.600	15.02	< 0.001
	Men	0.312	4.98	0.043
	All	0.352	11.39	0.001
	Women vs. Men	0.06	1.44	0.243
HR (bpm)	Women	0.873	68.8	< 0.001
	Men	0.828	52.8	< 0.001
	All	0.851	120.09	< 0.001
	Women vs. Men	0.128	3.09	0.094
CI (L/m^2^)	Women	0.450	8.17	< 0.001
	Men	0.479	10.1	< 0.001
	All	0.452	17.34	< 0.001
	Women vs. Men	0.046	1.01	0.326
SVI (ml/m^2^)	Women	0.874	69.5	< 0.001
	Men	0.878	79.1	< 0.001
	All	0.875	147.58	< 0.001
	Women vs. Men	0.005	0.114	0.739

*Partial Eta squared, F-statistics, and p-values comparing women and men with Type III sum of squares are reported in the last row for each parameter*.

### Central blood pressure reactions

Figure 2 details the central blood pressure reactions (MAP, DBP, and SBP) recorded during GIT in women and men. MAP in women was increased over baseline in P3 and P4 (*p* < 0.05), with MAP in P3 being higher than in P2 and P4 (*p* < 0.05). Throughout P6-P8, MAP was non-significantly increased over baseline in women. In men, MAP expressed a significant increase only during P6 and P8 (*p* < 0.01), while remaining non-significantly above baseline in all other phases. DBP in women was elevated throughout the first round (*p* < 0.01), and was higher during +2Gz phases than +1Gz phases (*p* < 0.001). During the second round, DBP increased over baseline only during P7 (*p* < 0.01). DBP in this phase was also higher than P6 and P8 (*p* < 0.01). DBP in men was significantly elevated over P1 during P3, P4, and throughout P6-P8 (*p* < 0.05), while DBP was higher during P3 than +1Gz phases (*p* < 0.05). SBP showed no significant changes from baseline in both genders. Men tended to have overall higher MAP, DBP, and SBP than women, however there were no significant gender differences found. Women did show a lesser degree of variability compared with men for MAP and DBP, particularly during +2Gz phases.

### Vascular resistance and cardiac reactions

Figure 3 details SVRI, HR, CI, and SVI reactions apparent during GIT in both genders. SVRI in women was significantly higher than baseline throughout all +Gz phases (*p* < 0.05), with SVRI tending to be higher in the +2Gz phases. However, there was no statistical difference between +1Gz and +2Gz. SVRI amongst men, although elevated during +Gz, was not significantly different from baseline. SVRI was the parameter that showed the highest variability in men, and this variability was markedly greater in men over women.

Women showed a significantly higher HR over baseline throughout all +Gz phases (*p* < 0.05), with significantly higher HR levels during +2Gz phases than +1Gz phases (*p* < 0.001). Men expressed significant HR elevations over baseline during P2, P3, and P7 (*p* < 0.05, *p* < 0.001, and *p* < 0.001). HR was also significantly higher during the +2Gz phases than +1Gz phases (*p* < 0.001). Women displayed non-significantly higher HR values over men throughout all phases of GIT (*p* = 0.094). CI showed no significant decreases from baseline amongst women, while in men, CI demonstrated significant decreases from P1 during P2, P4, and throughout P6-P8 (*p* < 0.05). SVI in both genders was consistently lower than baseline during all +Gz phases than baseline (*p* < 0.001), and lower in +2Gz than +1Gz (*p* < 0.001). Only in men, SVI was significantly increased over baseline during P5 (*p* < 0.05). Finally, no significant differences were found when comparing the two rounds of GIT with each other. Despite the gender specific differences, there was an overall similarity in cardiovascular reactions for men and women. Both HR and SVRI increased with higher +Gz levels, while SVI and CI displayed an inverse relationship with the +Gz level.

### Finisher women vs. womenNF

While a majority of women participating in this study (11/16) did not experience any orthostatic event during GIT, five experienced an orthostatic event and did not complete the full study protocol (womenNF). During the first minute of the initial +2Gz phase (P3), womenNF exhibited a significant decrease in MAP, SBP and SVI compared to baseline (*p* < 0.05), with no subsequent increases in HR and SVRI. Only two women went on to complete the protocol albeit at a reduced +Gz level (+1/1.5/1 Gz). A statistical comparison was performed between womenNF and finisher women during P2 and P3 (*n* = 5 vs. *n* = 11). Compared with finisher women, womenNF exhibited higher MAP, DBP, SBP, and SVRI during P2 (*p* < 0.05). During P3, the only difference between these groups was SVI, which was significantly lower in womenNF compared with finisher women (*p* < 0.05).

## Discussion

This study set out to determine gender specific cardiovascular reactions during intermittent +Gz interval protocol as a potential countermeasure against micro-g associated cardiovascular deconditioning. Upon conclusion of this study, it could be determined that women showed consistent significant increases in HR, and SVRI throughout the GIT, with significant DBP increases occurring during phases 2, 3, 4, and 7. On the other hand, men exhibited significant increases in DBP and HR only during the +2Gz phases with increases in DBP occurring in all but one +1Gz phase. No significant changes in SVRI for men were recorded. The lack of significant SVRI findings could be attributed to a high inter-group variability amongst the men involved in this study. SVRI in women did not show this extent of variation and responded in a uniform fashion. Amongst men, CI was significantly decreased throughout the GIT, while in women no significant changes were observed. As both genders had similar SVI reductions, this gender difference in CI is most likely due to the HR increases recorded amongst women over men. Finally, when comparing both rounds of the GIT, no significant differences were found. The cardiovascular reactions occurring in the first round were mirrored in the second round for both genders. This was the desired outcome of the interval protocol, and it can be assumed that a third round would have induced similar responses.

### Cardiovascular reactions during GIT: female specific reactions

To date, only few of studies dealt specifically with the topic of gender related effects of AG exposure. Two studies concluded that a single exposure to a graded AG protocol (from +0.6Gz to submaximal +Gz) significantly improved orthostatic tolerance amongst normovolemic and hypovolemic women (Evans et al., 2015; Goswami et al., 2015a). A third study found that 3 weeks of daily 35-min intermittent +Gz exposure coupled with exercise improved orthostatic tolerance amongst ambulatory women (Stenger et al., 2007). Other AG studies have focused on the pre-frontal cortical activity, which was decreased in women undergoing AG exposure with respect to men (Smith et al., 2013; Schneider et al., 2014). In the present study we aimed to investigate gender related differences in cardiovascular reaction during exposure to graded +Gz levels, while testing for the occurrence of a training effect during the second run. We found that women undergoing GIT exhibited significant increases in DBP, HR, and SVRI, which can be attributed to greater baroreflex sensitivity with respect to men during gravitational challenges (Hogarth et al., 2007). Another contributing factor for these findings could be plasma epinephrine, renin, and vasopressin levels, which have been recorded to be higher in women than in men are during gravitational challenges (Geelen et al., 2002). Although not measured in this study, increased plasma concentrations of the aforementioned neuroendocrine components could have led to the augmented SVRI, as well as HR activity. The low inter-individual variability displayed by women as a group would be advantageous when implementing future +Gz protocols, possibly negating the need to individualize a +Gz training profiles for women. A further reason for these reactions in women may be due to anthropometric factors, as a more compact body size in women may allow for increased sensitivity to gravitational challenges.

Although the majority of women (11/16) that participated in this study endured the GIT, five women did not follow suit and experienced an OI event. A predisposing factor for OI risk seems to be height and baseline central and systemic pressure, with taller women having higher MAP, DBP, SBP, and SVRI. This supports evidence that women have a higher propensity for OI during gravitational stress and the reasons for this have been discussed in prior research (Harm et al., 2001; Fu et al., 2004; Fong et al., 2007; Nordine et al., 2015) and indicate that anthropometric as well as physiological factors could play a role in this trend. It would appear that +2Gz exceeds the capacity for women with a certain height to maintain MAP, and a reduced +Gz profile would be beneficial. In order to ensure a 100% orthostatic tolerance rate amongst women, the +Gz limit for each person should be assessed prior to +Gz exposure, similar to the +Gz profile utilized by Goswami et al. (2015a). Alternatively, a modified +Gz profile could be used, as it appeared that 30% of women in this study could not tolerate full +2Gz for more than 60 s. These women may have benefitted from a starting +1Gz/+1.5Gz/+1Gz x2 profile.

### Cardiovascular reactions during GIT: male specific reactions

During GIT, men did not exhibit the same significant changes from baseline as women did. HR and DBP were significantly increased over baseline during +2Gz, but not SVRI. In men, a minimum of +2Gz seems to be needed in order to induce significant HR increases, a response which has also been recorded by Goswami et al., during SAHC training (Goswami et al., 2015b), Ueda et al. (2015) and Polese et al. (1992). A possible explanation as to why men require a stronger gravitational stimulus in order to trigger increases in HR may be due to higher resilience to venous pooling and central volume loss due to a greater amount of available plasma volume. Therefore, during +1Gz phases, while the decrease in functional blood volume is enough to trigger the beta sympathetic response in women, men may require a higher degree of SVI loss until HR increases become apparent. Surprisingly, SVRI in men showed no significant changes from baseline. However, as previously mentioned, this is more likely due to high inter-individual variability observed amongst men rather than low absolute values recorded. Possible explanations for these findings have been proposed by some study groups, which include polymorphic genetic differences in vascular resistance regulation (Seasholtz et al., 2006), or elevated respiratory effort during +Gz, which induces a higher amount of venous return to the central circuit (McKenzie, 1993). Although these factors may offer reasons for varying intensities of SVRI regulation, they do not offer insight to the difference in variability observed between genders, as these studies included exclusively men. In order to train both active vasomotor and cardiac mechanisms in men, a higher +Gz-level gradient may be required. For future GIT protocols in men, an individualized +Gz limit should be determined prior to training, as to maximize the effects on the cardiovascular system.

### Cardiovascular activity during GIT: gender differences

Although no significant gender differences in cardiovascular reactions were recorded, women tended to respond with heightened HR over men during the GIT. Other working groups have recorded similar HR responses in women compared to men during gravitational stress. These studies however used lower body negative pressure (LBNP) (Frey and Hoffler, 1988; Franke et al., 2000), and thus may not be directly comparable to SAHC studies. The gravitational vector applied by SAHC distributes a relatively equal transmural vascular pressure along the length of the body, whereas LBNP produces a strong transmural pressure directly below the LBNP seal (Dosel et al., 1998; Watenpaugh et al., 2004; Robertson, 2008).

The heightened HR response in women can be attributed to gender differences in the autonomic circuitry, myocardial structure, as well as hormonal status. Compared with men, women show increased parasympathetic withdrawal to the cardiac circuit during orthostatic stress thereby leading to an increase in HR (Evans et al., 2001; Huxley, 2007). In addition, the hormone estradiol may augment epinephrine sensitivity during orthostatic stress and thus be a contributing factor (Wenner et al., 2013; Gordon and Girdler, 2014). Further reasons for an increased HR in women could be differences in trans-mitral filling velocity and faster myocardial velocities (Nio et al., 2015). These factors would equate to a higher cardiac filling rate and faster myocardial contraction in women than in men, thus offering an account for differences in HR.

Regarding SVRI, no significant difference was found between genders, although men exhibited higher overall SVRI than women. This was reflected in higher MAP, DBP, and SBP recorded amongst men. Some groups have also observed significantly increased vasoconstrictor response in men over women during orthostatic challenges (Frey and Hoffler, 1988; Jarvis et al., 2010; Hachiya et al., 2012). However, more evidence suggests that peripheral vasoconstrictor activity does not differ between genders during orthostatic stress (Convertino, 1998; Franke et al., 2003; Kelly et al., 2004; Fu et al., 2005; Carter et al., 2015; Russomano et al., 2015; Patel et al., 2016). Certain authors have shown that during beta and muscarinic blockade, there is a predominant vascular regulation in men compared to dominant parasympathetic influence on heart rate regulation in women (Evans et al., 2001; Huxley, 2007). Those studies used different modalities to provoke orthostatic stress such as LBNP, head-up tilt (HUT), as well as pharmacological means. Previous studies that have recorded increases in vasomotor activity in men over women (Hachiya et al., 2012) have attributed these differences to an overall higher magnitude of sympathetic nerve activity to the periphery in men (Hart et al., 2009; Yang et al., 2012). It should be noted that SVRI is the total accumulation of vasoconstrictor activity in the circuit, which includes the extremities as well as the core (thorax, abdomen, and pelvis). Although not recorded in this study, studies have shown differences regarding vasomotor responses in different anatomical regions amongst men and women (Dart et al., 2002; Jarvis et al., 2010; Hachiya et al., 2012). Clearly, further AG studies are needed in order to critically examine gender differences regarding regional vascular activity during +Gz gradients via the use of Doppler sonography, laser Doppler, as well as near infrared spectroscopy.

## Limitations

While this study directly compared gender specific cardiovascular reactions during a newly devised graded intermittent AG training protocol, the findings should be interpreted with caution. Firstly, the small sample size used for this study, although of good size for human AG studies, makes drawing larger conclusions for the average population difficult. Also, as there is a lack of data concerning gender specific differences during SAHC exposure, more studies of this nature should be performed in order to ascertain if these gender specific reactions can be replicated. Moreover, this study did not set out to induce maximal +Gz limits in the human test subjects nor was the +Gz gradient tailored for each individual test subject. Had maximal +Gz profiles been deployed, or individual +Gz profiles been used, the results may have been different. Lastly, serum catecholamine, testosterone, estrogen, progesterone, and hemoglobin levels were not measured in this study. Thus, their role in the gender specific cardiovascular findings of this study can only be speculated upon. Additionally, no restrictions were placed on women concerning menstrual cycle phase, nor oral-contraceptive use upon partaking in this study. Certain authors have suggested that these factors may have an effect on autonomic functioning in women during gravitational stress (Wenner et al., 2013). In order to strengthen the findings of this study, more studies involving gender responses during +Gz exposure via SAHC, particularly with a similar +Gz profile, are warranted.

## Conclusions

This study showed that gender specific cardiovascular reaction patterns are indeed apparent during an intermittent AG session, while no significant differences between women and men were found. The gender specific cardiovascular differences in response to +Gz emphasize the importance of implementing AG countermeasure strategies specifically for women and men. Since micro-g induced cardiovascular deconditioning leads to vascular dysregulation upon reintroduction of standard gravity (Waters et al., 2002), a potential countermeasure protocol should ensure adequate vasomotor stimulation in both genders. This GIT, which lasted for 45 min, consistently stimulated vasomotor activity amongst women with SVRI increasing in all phases and DBP during the +2Gz phases. Men may require higher +Gz gradients in order to exhibit similar reactions. Also, only 70% of women finished the entire +Gz exposure. In order to ensure a 100% tolerability rate as well as ensuring the desired cardiovascular reactions needed to overcome orthostatic instability upon re-introduction of gravity modified or individualized +Gz profiles should be deployed, as was demonstrated by previous AG studies (Goswami et al., 2015a). Also, further testing in conjunction with bed-rest, immersion, and isolation studies are needed to see if this GIT training can mitigate cardiovascular deconditioning prior to manned space flight implementation.

## Author contributions

ZM and MN wrote the manuscript and analyzed the data with the help of KB, BH, SM, and AW. KB, AW, BH, SM, and MN performed statistical analysis. AS and OO implemented the data collection. HH, H-CG and OO conceived and planned the study. MM, HH, H-CG, OO, AW, SM, and KB provided expertise, reviewed, and approved the final manuscript.

### Conflict of interest statement

The authors declare that the research was conducted in the absence of any commercial or financial relationships that could be construed as a potential conflict of interest.
